# Conserved Role for Biofilm Matrix Polysaccharides in *Candida auris* Drug Resistance

**DOI:** 10.1128/mSphereDirect.00680-18

**Published:** 2019-01-02

**Authors:** E. G. Dominguez, R. Zarnowski, H. L. Choy, M. Zhao, H. Sanchez, Jeniel E. Nett, D. R. Andes

**Affiliations:** a Department of Medicine, Section of Infectious Disease, University of Wisconsin—Madison, Madison, Wisconsin, USA; b Department of Medical Microbiology and Immunology, University of Wisconsin—Madison, Madison, Wisconsin, USA; University at Buffalo; Albert Einstein College of Medicine; University of Tennessee at Memphis

**Keywords:** *Candida auris*, biofilm, matrix, resistance

## Abstract

Candida auris has emerged as an outbreak pathogen associated with high mortality. Biofilm formation and linked drug resistance are common among *Candida* species. Drug sequestration by the biofilm matrix accounts for much of the antifungal tolerance. In this study, we examine the biofilm matrix composition and function for a diverse set of C. auris isolates. We show that matrix sequesters nearly 70% of the available triazole antifungal. Like the biofilms formed by other *Candida* spp., we find that the matrix of C. auris is rich in mannan-glucan polysaccharides and demonstrate that their hydrolysis reduces drug tolerance. This biofilm matrix resistance mechanism appears conserved among *Candida* species, including C. auris.

**IMPORTANCE** Candida auris is an emerging fungal threat linked to poor patient outcomes. The factors responsible for this apparent increase in pathogenicity remain largely unknown. Biofilm formation has been suggested as an important factor for persistence of this organism in patients and the environment. Our findings reveal one mechanism utilized by C. auris to evade the effect of triazole antifungal therapy during biofilm growth. The conservation of the protective biofilm matrix among *Candida* spp. suggests that is a promising pan-fungal *Candida* biofilm drug target.

## Abstract (precis)

Candida auris is an emerging fungal threat linked to poor patient outcomes. The factors responsible for this apparent increase in pathogenicity remain largely unknown. Biofilm formation has been suggested as an important factor for persistence of this organism in patients and the environment. Our findings reveal one mechanism utilized by C. auris to evade the effect of triazole antifungal therapy during biofilm growth. The conservation of the protective biofilm matrix among *Candida* spp. suggests that is a promising pan-fungal *Candida* biofilm drug target.

## INTRODUCTION

Candida auris has recently emerged as a significant nosocomial pathogen (1–4). The organism exhibits several concerning features compared to other *Candida* species, including the capacity for persistent colonization of nosocomial surfaces and frequent resistance to antifungal drugs. Outcomes of infection have generally been poor, with mortality rates near 60% in some case series (2). Due to its recent emergence, relatively little is known regarding the virulence attributes of this pathogen.

The isolation of C. auris from wounds, catheters, and the hospital environment suggests the involvement of biofilms (5, 6). These surface-associated communities exhibit adaptive antimicrobial resistance, and their presence during infection contributes to persistence and excess mortality (7–9). While *Candida* species possess diverse mechanisms to defend against antifungal threats during biofilm growth, the extracellular matrix encasing the organisms accounts for a significant portion of the observed drug tolerance (8, 10, 11) This material sequesters antifungals, preventing them from reaching their cellular targets (10, 11). For Candida albicans, Candida glabrata, Candida tropicalis, and Candida parapsilosis, a matrix carbohydrate complex composed of mannan and glucan is closely linked to this mechanism of biofilm resistance (11–13). We questioned if biofilms formed by C. auris may exhibit resistance through a similar mechanism. Here, we explore biofilm-associated drug tolerance, matrix composition, and matrix function for a diverse collection of C. auris isolates. The strains are representative of the genetically distinct clades that have arisen throughout the world (2).

## RESULTS

### *C. auris* isolate biofilm formation, drug tolerance, and antifungal sequestration.

**FIG 1:**
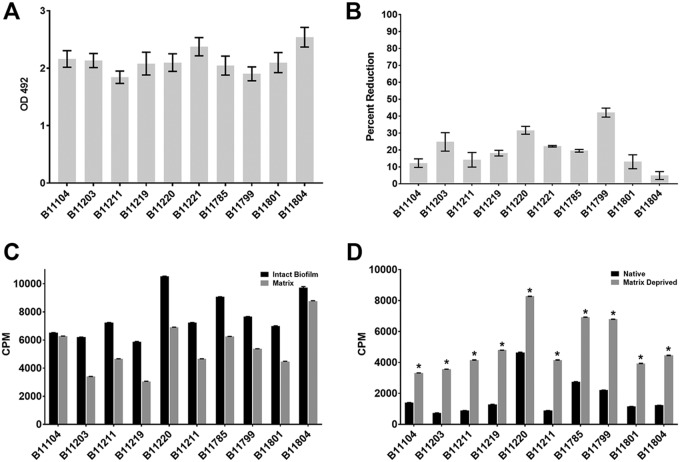
C. auris biofilm, drug susceptibility, and antifungal sequestration. (A) Biofilm formation was assessed using an XTT assay in a 96-well polystyrene plate assay after a 24-h incubation. (B) Biofilm antifungal susceptibility following 24 h of treatment with 1,000 μg/ml of fluconazole compared with untreated biofilms. Biofilm reduction was assessed using an XTT assay in a 96-well polystyrene plate assay and is reported as a percentage of reduction compared to untreated control wells. (C) Sequestration of ^3^H-labeled fluconazole was assessed using *in vitro* intact biofilms and the isolated extracellular matrix. Results are expressed as counts per minute (CPM). (D) Sequestration of ^3^H-labeled fluconazole was assessed inside cells (intracellular) after isolation from biofilms with and without (matrix eliminated) extracellular matrix. Extracellular matrix was removed physically by sonication. Results are expressed as counts per minute (CPM). The asterisks indicate statistically significant differences (*P* < 0.0001) between matrix-deprived biofilms and intact biofilms containing matrix based upon unpaired two-tailed *t* test.

**FIG 2:**
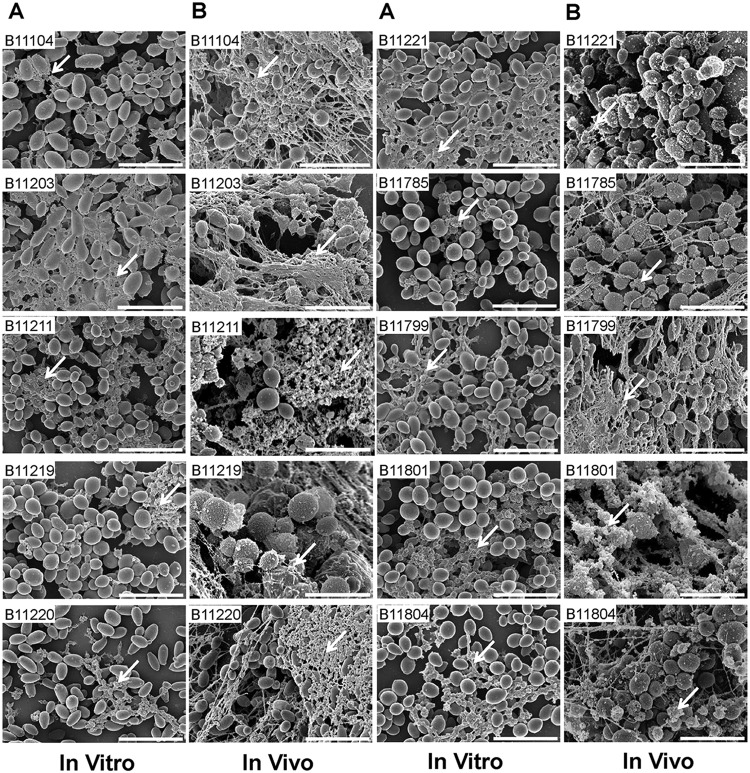
C. auris biofilm ultrastructure. (A) Biofilm architecture from *in vitro* coverslips was assessed using SEM after 24 h of incubation. (B) Biofilm formation was investigated on the intraluminal catheter surface from the *in vivo* rat central venous catheter model. All biofilms were assessed visually using SEM imaging after 24 h of incubation.

Because circulating C. auris strains vary genetically, we examined biofilm growth for a set of 10 isolates, including each of the common clades (Table 1). We utilized complementary *in vitro* and *in vivo* assays to assess the capacity of each strain to form biofilm (14, 15). To quantify *in vitro* biofilms, we grew biofilms in microtiter plates and estimated the burdens by metabolic activity using an XTT [2,3-bis-(2-methoxy-4-nitro-5-sulfophenyl)-2H-tetrazolium-5-carboxanilide salt] assay. Biofilm growth was similar among the strains (mean ± standard deviation optical density at 492 nm [OD_492_], 2.13 ± 0.20; range, 1.84 to 2.54) (Fig. 1A). To assess the architecture of each biofilm, we utilized scanning electron microscopy. We examined biofilms formed *in vitro* on coverslips and *in vivo* using a rat catheter model (Fig. 2). We observed moderate variation among the strains. Each isolate formed biofilms marked by an accumulating strata of yeast forms. Extracellular matrix coated the fungal cells and was most evident in the *in vivo* model, as previously described (16).

**TABLE 1:** C. auris geographic clade and planktonic MIC

Strain	Country of origin	Planktonic fluconazole MIC (μg/ml)
B11104	Pakistan	256
B11203	Colombia	256
B11211	India	256
B11219	India	256
B11220	Japan	4
B11221	South Africa	128
B11785	Colombia	8
B11799	Colombia	16
B11801	Colombia	16
B11804	Colombia	2

A 96-well *in vitro* biofilm assay was used for assessment of antifungal (fluconazole) susceptibility using an XTT endpoint (17). Following exposure to a maximal concentration of fluconazole of 1,000 μg/ml (based upon solubility), the majority of cells in the biofilm communities remained viable (or metabolically active) (Fig. 1B). Fluconazole did not produce a 50% reduction for any of the strains, including those susceptible to fluconazole during planktonic growth. (Previously reported planktonic MICs are reported in Table 1.) We observed a modest degree of variability in response (range, 4.94 to 42.1%). We considered this may represent the involvement of intrinsic resistance mechanisms for individual strains, as the planktonic MICs varied 128-fold. However, we found no relationship between planktonic MICs and the activity of fluconazole against biofilms, underscoring the biofilm-specific nature of the resistance observed in the present studies.

Previous studies with other *Candida* species have demonstrated drug sequestration by the biofilm-encasing extracellular matrix (11, 13). To determine if the extracellular matrix of C. auris biofilms may similarly sequester antifungals, we tracked radiolabeled fluconazole following administration to C. auris biofilms (Fig. 1C and D). We found that the majority of fluconazole within the biofilm was retained in the extracellular matrix (Fig. 1C; mean ± standard deviation, 69% ± 14%; range, 52 to 90%). This is consistent with antifungal sequestration and similar to investigations with other common *Candida* species (13).

Physical removal of extracellular matrix via sonication has been a useful laboratory method to assess the impact of matrix drug sequestration (11, 13). Elimination of matrix for each of the C. auris isolates increased the intracellular accumulation of fluconazole by more than 3-fold (Fig. 1D; mean ± standard deviation 3.47 ± 1.01-fold increase in susceptibility compared to fluconazole alone; range, 1.78 to 4.87-fold; *P* < 0.0001).

### *C. auris* biofilm extracellular matrix composition and function.

**FIG 3:**
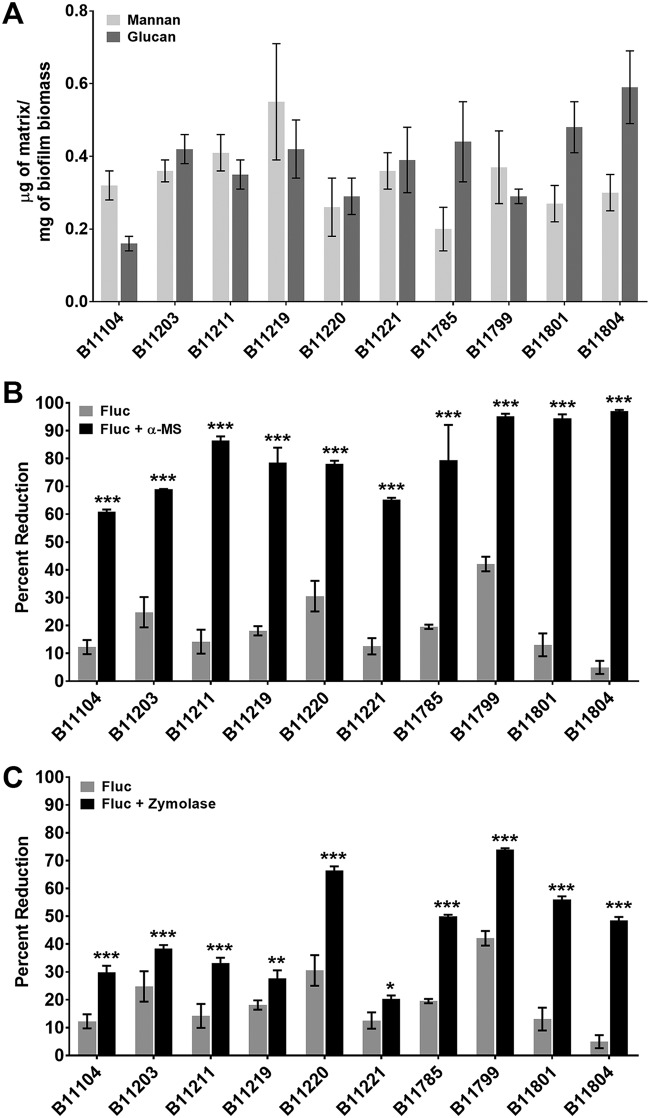
C. auris biofilm extracellular matrix composition and function. (A) Concentration of mannan and glucan in the extracellular matrix of ten C. auris isolates after 24 h of incubation in a 6-well polystyrene format using gas chromatography. (B) Biofilms were treated with fluconazole (Fluc; 1,000 μg/ml) with or without mannan hydrolysis using α-mannosidase (α-MS). Efficacy was assessed in a 96-well polystyrene format using XTT to assess biofilm cell metabolic activity. The asterisks indicate statistically significant differences (*P* < 0.001) between combination therapy and fluconazole monotherapy based upon ANOVA using the Holm-Sidak method for pairwise comparison. (C) Biofilms were treated with fluconazole (1,000 μg/ml) with or without glucan hydrolysis using zymolyase. Efficacy was assessed in a 96-well polystyrene format using XTT to assess biofilm cell metabolic activity. The asterisks indicate statistically significant differences (*, *P* = 0.02; **, *P* = 0.004; ***, *P* < 0.001) between combination and fluconazole monotherapy based upon ANOVA using the Holm-Sidak method for pairwise comparison.

A polysaccharide complex of mannan and glucan is a signature component of the biofilm extracellular matrix of several *Candida* species (11–13). This mannan-glucan complex has been linked to biofilm drug resistance through a mechanism of antifungal sequestration (11). To evaluate for a similar complex in C. auris biofilms, we isolated extracellular matrix and analyzed the polysaccharide by gas chromatography. Each of the biofilms contained both mannan and glucan polymers (Fig. 3A). However, the content of the polymers was somewhat variable, as was the ratio of mannan and glucan, but on average, the amounts were similar (mean ± standard deviation ratio of mannan to glucan, 1.0 ± 0.47).

We next explored a role for matrix mannan and glucan in biofilm drug resistance pharmacologically via hydrolysis of the individual polysaccharides (Fig. 3B and C). We first pretreated biofilms with either mannosidase or glucanase to disrupt the corresponding matrix components. We subsequently performed fluconazole susceptibility testing on the biofilms (11, 13). We found that the destruction of each matrix polysaccharide enhanced fluconazole susceptibility for all C. auris biofilm isolates. Treatment with mannosidase resulted in an ∼60% decrease in biofilm burden following fluconazole exposure (mean ± standard deviation 61.2% ± 15.9% difference in biofilm reduction; range, 44.1 to 92.2%). Similarly, hydrolysis of matrix glucan also impacted the antifungal biofilm effect, but to a lesser degree, with an average biofilm burden reduction of ∼25% (mean ± standard deviation 25.2% ± 13.4% difference in biofilm reduction; range, 9.6 to 43.5%). These observations support a role for matrix mannan and glucan in antifungal sequestration and biofilm drug resistance, as has been described for other *Candida* species.

## DISCUSSION

C. auris is the first fungal species designated as a global outbreak pathogen, and national reporting will be required by the CDC beginning in 2019 (1–4). Associated drug resistance and high mortality have hastened pathogenesis investigation (5, 18). However, fungus-derived attributes responsible for these clinical phenomena remain largely unknown. One virulence factor that is arguably universal among *Candida* species is the ability to thrive in biofilm communities protected from therapeutics and the immune system. Recent studies have illustrated the ability of C. auris to produce biofilms, resist therapeutics, and evade host defenses (5, 19, 20).

*Candida* species possess a diverse biofilm tool kit that provides a multipronged defense against antifungal threats (21, 22). A major degree of resistance is afforded by the biofilm extracellular matrix. The present investigations demonstrate that C. auris exploits similar tools for biofilm community persistence. However, other factors may contribute to biofilm drug resistance. It is intriguing to speculate that involvement of alternative mechanisms may account for the variation we observed in drug resistance among the C. auris strains. In an effort to uncover mechanisms linked to drug resistance, Kean et al. examined transcript profiling of C. auris biofilms (23). Similar to study in C. albicans, they identified an increase abundance of efflux pump transcript, suggesting a role in resistance (21). Interestingly, they also observed elevated expression of several glucan-modifying genes of demonstrated relevance for the matrix drug sequestration phenomenon (10, 23). The biochemical and functional matrix observations in the present studies are congruent with results from other species. The matrix and other resistance mechanisms should be further explored for identification of novel biofilm drug targets.

## MATERIALS AND METHODS

### Strains.

Ten C. auris isolates were obtained from the Centers for Disease Control and Prevention (Table 1).

### *In vitro* and *in vivo* biofilm imaging.

*In vitro* biofilms were grown on coverslips in 6-well polystyrene plates. Ten microliters of fetal calf serum (FCS) was placed on each coverslip and dried for 1 h. Forty microliters of an inoculum of 10^8^ cells/ml was placed on the treated coverslip and incubated at 37°C for 24 h with orbital shaking at 50 rpm. Following incubation, the cells were fixed with 4% (vol/vol) formaldehyde and 1% glutaraldehyde at 4°C overnight. Coverslips were then washed with PBS and treated with 1% osmium tetroxide for 30 min at 22°C. Samples were subsequently washed with a series of increasing ethanol dilutions (30 to 100% [vol/vol]), followed by critical point drying and coating with platinum. Scanning electron microscopy (SEM) of samples was performed using a LEO 1530 microscope.

*In vivo* biofilms were propagated in a rat central venous catheter biofilm model as previously described (11). After a 48-h biofilm formation phase, the devices were removed, sectioned to expose the intraluminal surface, and processed for SEM imaging identically as described above.

### *In vitro* biofilm quantification and antifungal susceptibility testing.

Biofilm quantification and susceptibility assays were conducted on biofilms grown in 96-well polystyrene plates. C. auris (100 μl at 10^8^ cells/ml) was added to each well and incubated statically for 24 h at 37°C. For experiments examining susceptibility, fresh media and the antifungal and/or enzymes (1000 μg/ml) were added after the 24 h of incubation and incubated for an additional 24 h. Reagents included α-mannosidase (0.78 U/ml, jack bean; Sigma), zymolyase (0.63 U/ml; MP Biomedicals), and fluconazole (1,000 μg/ml). Biofilms were quantified using a tetrazolium salt XTT reduction assay. Briefly, media and nonadherent cells were removed, and biofilms were washed with sterile PBS. XTT (80 μl; 0.75 mg/ml), phenazine methosulfate (PMS) (10 μl; 320 μg/ml), and 10 μl of 20% glucose were added, and plates were incubated for 60 min at 37°C in the dark. Absorbance at 492 nm was measured using an automated plate reader. Biofilm reduction was calculated by comparing untreated biofilms with those treated. Assays were performed in triplicate, and differences were assessed by analysis of variance (ANOVA) with pairwise comparisons using the Holm-Sidak method.

### Sequestration of [^3^H]fluconazole in biofilms.

Radiolabeled fluconazole (50 μM, 0.001 mCi/ml in ethanol; Moravek Biochemicals) was utilized to measure drug retention in biofilms (10). Biofilms were grown for 48 h in 6-well plates as described above. Briefly, an inoculum of 10^8^ cells/ml was added to each well. Biofilms were grown at 37°C for 48 h on an orbital shaker set at 50 rpm. After the 48 h of incubation, medium was removed, and biofilms were washed with sterile water and then incubated with 8.48 × 10^5^ cpm of [^3^H]fluconazole in RPMI-MOPS (morpholinepropanesulfonic acid) for 30 min at 37°C. Unlabeled fluconazole (20 μM) in RPMI-MOPS was added for an additional 15-min incubation period. After a second wash, biofilms and matrix were collected and isolated, as described above. An aliquot of each intact biofilm was collected for scintillation counting. The remaining cells were then disrupted by bead beating to yield cell wall and intracellular portions. To quantify the radiolabeled fluconazole in each component, samples were analyzed using a Tri-Carb 2100TR liquid scintillation analyzer after the addition of ScintiSafe 30% liquid scintillation counting (LSC) mixture. Three replicates were averaged, and values were compared to those of the reference strain.

### Biofilm matrix isolation and analysis.

Biofilms were grown in 6-well polystyrene plates as described above, and extracellular matrix was collected from mature 48-h biofilms as previously described (10, 11). Briefly, biofilms were removed with a spatula and harvested in sterile water. Biofilms were then sonicated for 20 min, and matrix was separated from the biomass by centrifugation of the samples at 2,880 × *g* for 20 min at 4°C. To determine the concentration of mannan and glucan within the matrix, sugars were quantified by gas-liquid chromatography–flame ionization detector (GLC-FID) on a Shimadzu GC-2010 system after conversion to alditol acetate derivatives as previously described (12). Data for these monosugars were calculated and are presented as micrograms of matrix per milligram of biofilm biomass.
